# Unsupervised machine learning identifies opioid taper reversal patterns in a longitudinal cohort (2008–2018)

**DOI:** 10.1371/journal.pdig.0000785

**Published:** 2025-04-07

**Authors:** Monika Ray, Joshua J. Fenton, Patrick S. Romano

**Affiliations:** 1 Department of Internal Medicine, School of Medicine, University of California Davis, Davis, California, United States of America; 2 Center for Healthcare Policy and Research, University of California Davis, Davis, California, United States of America; 3 Department of Family and Community Medicine, School of Medicine, University of California Davis, Davis, California, United States of America; ZEW - Leibniz Centre for European Economic Research: ZEW -Leibniz-Zentrum fur Europaische Wirtschaftsforschung GmbH Mannheim, GERMANY

## Abstract

Chronic pain is commonly treated with long-term opioid therapy, but rapid opioid dose tapering has been associated with increased adverse events. Little is known about heterogeneity in the population of patients on high dose opioids and their response to different treatments. Our aim was to examine opioid dose management and other patient characteristics in a longitudinal, clinically diverse, national population of opioid dependent patients. We used spectral clustering, an unsupervised artificial intelligence (AI) approach, to identify patients in a national claims data warehouse who were on an opioid dose tapering regimen from 2008-2018. Due to the size and heterogeneity of our cohort, we did not impose any restrictions on the kind or number of clusters to be identified in the data. Of 113,618 patients with 12 consecutive months at a stable mean opioid dose of  ≥ 50 morphine milligram equivalents, 30,932 had one tapering period that began at the first 60-day period with  ≥ 15% reduction in average daily dose across overlapping 60-day windows through 7 months of follow-up. We identified 10 clusters that were similar in baseline characteristics but differed markedly in the magnitude, velocity, duration, and endpoint of tapering. A cluster comprising 42% of the sample, characterised by moderately rapid, steady tapering, often (73%) to a final dose of zero, had excess drug-related events, mental health events, and deaths, compared with a cluster comprising 55% of the sample, characterised by slow, steady tapering. Four clusters demonstrated tapers of various velocities followed by complete or nearly complete reversal, with combined drug-related event rates close to that of the slowest tapering cluster. Unsupervised AI methods, such as spectral clustering, are powerful to identify clinically meaningful patterns in opioid prescribing data and to highlight salient subpopulation characteristics for designing safe tapering protocols. They are especially useful for identifying rare events in large data. Our findings highlight the importance of considering tapering velocity along with duration and final dose and should stimulate research to understand the causes and consequences of taper reversals in the context of patient-centered care.

## Author summary

Large data warehouses, such as those developed by insurance agencies, allow for population health research which is generalisable. However, due to the enormity and heterogeneity of the data, many hidden or unsuspected patterns are overlooked. These underlying patterns are important because they not only aid in a deeper understanding of the inferences from hypothesis testing but also uncover new testable hypotheses. In our analyses, we show an application of unsupervised machine learning to identify hidden patterns in large data. Our aim was to use methods that did not impose any restrictions on the kind of patterns to be identified nor bias the approach to lead us to results that are well studied in this domain, i.e., we were searching for novel, unreported characteristics. Since large datasets also contain noise, we applied a true data “mining” approach where one can choose to take what is valuable and throw away the rest. Our results using spectral clustering identified clinically meaningful patterns, including rare events such as taper reversals. Our application of clustering to opioid dose tapering is an innovative approach of highlighting new hypotheses to study and also an example of how unsupervised machine learning methods can be used to discover rare events as they do not follow normal distributions. Powerful unsupervised pattern recognition methods can be applied to other population health studies performed on large data to advance scientific knowledge.

## Introduction

Chronic pain is a debilitating condition that affects over 51 million Americans, or nearly 21% of the adult population in 2021 [1]. About 22% of adults with chronic pain in the U.S. have used a prescription opioid in the past 3 months, with higher use (26%) among those with less than a high school education [2]. Although opioid analgesics reduce pain and improve physical functioning in patients with chronic non-cancer pain [3], these benefits are modest and must be balanced against significant risks [4]. In 2022, 14,716 Americans died from overdoses involving prescription opioids, and many more died from illicit opioids after becoming addicted to prescription products [5,6].

National prescribing guidelines have led to substantial dose tapering among patients on long-term opioid therapy for chronic pain, especially since 2016 [7,8]. A widely used quality metric for health plans and physicians encourages opioid doses below 90 morphine milligram equivalents (MME) per day [9,10]. Several studies have shown worse outcomes associated with rapid dose reduction [11–13] but the underlying heterogeneity of tapering patterns and outcomes among these patients with chronic non-cancer pain has not been explored. Opioid-dependent patients with chronic pain often resist any dose reduction [14], while pharmacies and regulators encourage dose reduction. To inform better clinical practice, we need to understand how the peak tapering velocity fits into overall patterns of opioid dose management and explore the characteristics of higher- and lower-risk patients.

Unsupervised machine learning (ML) is a form of AI where a model highlights patterns in the data without any explicit feedback, i.e., the outcome variable (label) is not specified nor is the model assessed with respect to it. Unsupervised ML can be used to identify patient subpopulations without imposing any assumptions on the patterns that emerge. The purpose behind this is to identify patterns that a human wouldn’t know existed and, thus, never would have designed a cohort or model to account for (e.g., stratified analyses). Pattern recognition approaches are powerful for identifying novel patterns that usually get missed in hypothesis driven research [15]. Our research is focused on highlighting the need to use unsupervised ML on extremely large datasets, such as large insurance-based cohorts, that usually violate assumptions used in most supervised and parametric algorithms. Spectral clustering is a well-established method that has been applied in various biomedical applications; our innovation is to apply it to opioid dosing data [16–18]. First we use the clustering algorithm to group phenotypes into clusters, without classifying variables as independent or dependent, and then identify characteristics within these clusters [19].

### Materials and methods

### Data

We obtained data from 2008-2018 for adults from the OptumLabs Data Warehouse (OLDW), which contains de-identified administrative claims data, including medical and pharmacy claims and eligibility information for about 120 million enrollees, representing a mixture of ages and regions across the United States. This cohort created by Agnoli and colleagues had a stable baseline period of 12 consecutive months at an opioid dose  ≥ 50 MME [11]. The tapered cohort was defined as patients who had a dose tapering phase, which began on the first 60-day period with  ≥ 15% reduction in average daily dose across overlapping 60-day windows through the initial seven months of follow-up. Patients who had  ≥ 15% reduction in average daily dose over a longer time frame were not included due to uncertainty about the intent of slight MME dose reductions (which could be driven by delays in picking up prescriptions). To facilitate interpretation, we selected patients who had only a single period of tapering during the study period. Mortality in the tapered cohort was determined by analysing the time after taper initiation and matching against the OLDW mortality table.

Adverse events included emergency department (ED) visits or hospital admissions for (1) drug or alcohol overdose or withdrawal (drug-related events); and (2) depression or suicide attempts or intentional self-harm (mental health events). These events were identified using International Classification of Diseases, Tenth/Ninth Revision, Clinical Modification (ICD-10-CM/ICD-9-CM) diagnosis codes [11]. Comorbidities were identified using the AHRQ Elixhauser Comorbidity Software [20]. This project was determined by the University of California Office of the President to be exempt from human subjects review, as the OptumLabs Data Warehouse (OLDW) uses completely de-identified, anonymised data.

### Analytic methods

We used a novel spectral clustering algorithm (Spectrum) [21]. Spectral graph theory associates the spectrum of a matrix, i.e. eigenvalues of a matrix, to the properties of a graph via the Laplacian matrix [22,23]. It operates on graphs that are constructed between neighbouring nodes that represent data points (i.e., patients). It identifies arbitrarily shaped clusters (with convex or non-convex boundaries) using the eigenvectors [18,24–26]. A Laplacian similarity matrix models the local neighborhood relationships between data points as an undirected graph [27–29]. Spectral clustering is robust to the geometry of the clusters and outliers, and does not require the user to specify the number of clusters [30–32]. Other clustering methods, hierarchical clustering and decision trees, were considered but were not well suited for our research aims. For example, K-means clustering is a popular clustering algorithm based on many restrictive assumptions, which real-world datasets often violate [33,34]. It operates on the input data matrix and, hence, is sensitive to the size of data and number of features. Group-based trajectory modelling (GBTM) is a method used to group cases in a dataset based on their non-monotonic trajectories, but it makes strong assumptions on the distribution of trajectories within groups [35–37]. However, these algorithms are widely used due to their availability in standard statistical programming languages and ease of implementation, but they are not appropriate for exploratory analyses of complex, multidimensional data with an unknown number of clusters.

The spectral clustering pipeline involves (1) creation of the similarity matrix, then (2) creation of the Laplacian matrix, and finally (3) creation of clusters [26,38]. Variations of spectral clustering algorithms address issues related to creation of the similarity matrix, graph-partitioning and speed on massive datasets. Since spectral clustering operates on the Laplacian similarity matrix, it is sensitive to the data size. The algorithm developed by John and colleagues is novel in the way it combines use of the following features: (1) Zelnik-Manor’s self-tuning [39] and Zhang’s density-aware [40] kernels to create the similarity matrix, (2) Ng’s spectral clustering method to estimate the optimal number of clusters [18] and Gaussian mixture modelling (GMM) [41] to cluster the data, and (3) a fast approximate spectral clustering (FASP) method [42] to allow for fast clustering of massive data on regular machines. The self-tuning component of the kernel adjusts to the scale of the data, while the density-aware component adapts to the local density of the data, creating more or fewer connections depending on the density of the regions. Spectrum uses the diffusion of tensor product graphs (TPG) to capture higher order information in the data and highlight underlying patterns in the data [43]. The final clusters are plotted using the first two principal components, PC1 and PC2. We did not use the eigen gap-statistic to determine the number of clusters as it was not essential for us to constrain the number or size of clusters. Furthermore, the eigen gap heuristic to force the number of clusters works well if there are well-defined clusters but not when there are noisy or overlapping clusters, which is a reasonable assumption in all real-world datasets in healthcare. [26,44,45].

The variables provided to the clustering algorithm were age at taper initiation (base_age), sex (female), average opioid dose by month (avg_m), mean baseline dose (avg_base) and the number of months from baseline to taper initiation (numMontoTapr), counts of drug-related (OD_cnt_) and mental health events (MH_cnt_) in the pre-taper and post-taper phases, benzodiazepine co-prescription at baseline (copres_benzo_0) and at 30 days (copres_benzo_m30), and 31 Elixhauser comorbidity variables. For counterfactual inference, we identified the number and proportion of adverse events in each cluster, and then computed the excess number of those events relative to the null assumption of equal event risk across all clusters, whose equation is shown here -


$$\begin{array}{rcll} ExcessEvents=(NumEventsCluster)-(NumPatientsCluster*(\frac{TotalEvents}{TotalPatients})) & & & \end{array}$$

where:

*ExcessEvents* = number of excess events in cluster
*NumEventsCluster* = number of observed events within cluster
*NumPatientsCluster* = number of patients in cluster
*TotalEvents* = total number of adverse events in cohort
*TotalPatients* = total number of patients in cohort

## Results

**Fig 1:**
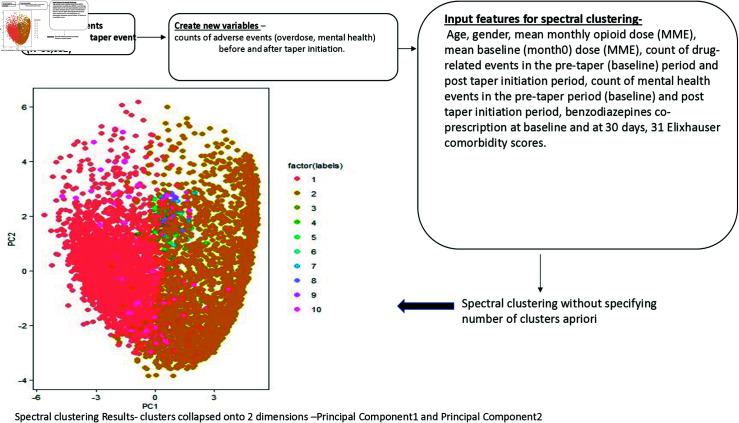
Analysis flowchart. The clustering plot is shown with data collapsed onto 2-dimensions represented by principal component 1 (x-axis) and principal component 2 (y-axis).

33,628 patients in our cohort had one or more phases of opioid dose tapering based on the tapering definition of  ≥ 15% reduction in average daily dose in 7-months of follow-up [11]. Spectral clustering of 30,932 patients who underwent single tapers resulted in 10 clusters (subpopulations). Fig 1 shows the analytical pipeline and the plot of these clusters. We could not clearly show all ten clusters as the plots are created by collapsing them onto the first two principal components. Fig 1 shows that the clusters are not spherical and the data have outliers. Table 1 shows the characteristics of patients who tapered.

**Table 1:** Characteristics of entire cohort (N = 33,628) who tapered.

Variables	Categories	n (%)
Gender	Female	18,197 (54.11)
	Male	15,431 (45.89)
Number of Tapers	1	30,932 (91.98)
	2	2,462 (7.32)
	> = 3	234 (0.70)
Number of drug-related events before tapering	0	32,238 (95.87)
	1	1,182 (3.51)
	> = 2	208 (0.62)
Number of drug-related events after tapering	0	31,210 (92.81)
	1	1,888 (5.61)
	2	356 (1.06)
	> = 3	174 (0.52)
Number of mental health events before tapering	0	14,788 (43.98)
	1	3,984 (11.85)
	2	2,949 (8.77)
	3	2,040 (6.07)
	4	1,665 (4.95)
	5	1,223 (3.64)
	6	1,034 (3.07)
	> = 7	5,945 (17.68)
Number of mental health events after tapering	0	32,041 (95.28)
	1	1,096 (3.26)
	2	300 (0.89)
	> = 3	191 (0.57)
Number of mental health events after tapering	0	32,041 (95.28)
	1	1,096 (3.26)
	2	300 (0.89)
Age	**Mean ± Std.**	58.0 ± 11.6

In order to understand patient heterogeneity, we need to study cluster differences using the features present in the data. We have estimated 95% confidence intervals surrounding all estimates of adverse event rates by cluster. These confidence intervals provide statistical support for the argument that the most populated clusters have different patterns of subsequent adverse events. Null hypothesis significance testing (i.e., p values) would be potentially misleading because our analysis did not have pre-specified hypotheses and the American Statistical Association advises against reporting p values in this situation [46–48]. All clusters had patients with high mean baseline doses of 140-237 MME/day. Of particular interest were the three large clusters and their baseline characteristics, shown in Table 2. These large clusters (1, 2, and 10) were very similar demographically (e.g., age, sex, rurality, imputed education and race), on baseline co-prescribing of benzodiazepines, and on comorbid diagnoses during the baseline year, such as alcohol abuse and dependence, drug abuse and dependence, and depression. They had similar medical experiences during their pre-taper period of stable opioid dosing, with relatively few drug-related events (mean 0.04, 0.05, and 0.04, respectively) and more mental health events (mean 3.81, 4.03, and 3.66, respectively). The most notable characteristic that distinguished clusters was the dose tapering pattern, especially the trajectory and final opioid dose, so we examined these features in detail.

**Table 2:** Patient characteristics (N = 30,932) in cluster cohorts before taper initiation.

Cluster	No. patients (%)	Age (Mean)	Female (%)	benzodiazepines Rx (%)	Alcohol abuse (%)	Depression (%)	Drug abuse (%)	Drug-related Health event counts (Mean)	Mental event counts(Mean)	Base dose (Mean MME)
1	16,965(54.8)	58.7	55.7	28.9	2.4	31.7	16.6	0.04	3.81	189.8
2	13,025(42.1)	57.0	53.1	30.1	3.0	31.4	16.5	0.05	4.03	192.3
10	531(1.7)	58.4	49.5	29.7	3.4	30.3	15.1	0.04	3.66	140.3
3 - 5*	185( < 1%)	58.6	49.2	32.4	3.2	33.5	21.6	0.04	4.01	209.9
6 - 9*	226( < 1%)	57.6	41.2	31.0	2.7	27.9	16.4	0.04	3.73	219.1

*Clusters 3-5 and clusters 6-9: values for these clusters are combined to adhere to small cell size policy.

*Weighted mean calculated for these clusters.

**Fig 2:**
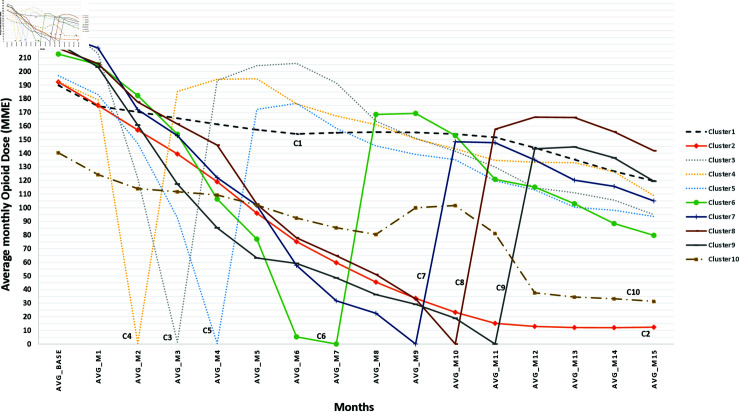
The average monthly dose in MME for all the patients within each cluster.

Fig 2 graphs the tapering trajectories of the three large clusters (See S1 Table). Each trajectory is plotted as the average monthly dose of the patients in that cluster. The largest clusters had markedly different opioid dose tapering trajectories and associated adverse events as shown in Table 3. The estimated number of excess events represents the difference between the number of observed events and the number of events that would have occurred if all clusters had the same event rate. About 55% of patients were in cluster 1, characterised by very slow and steady tapering to a final dose about two-thirds of baseline, with low event rates and no reversal. While clusters 2 and 10 looked quite similar in their baseline characteristics, they had very different taper trajectories. Cluster 2 was characterised by relatively rapid tapering to zero or very low doses, while cluster 10 was characterised by somewhat slower tapering from lower mean baseline doses (140.3 versus 192.3 MME)to higher mean end doses (37.6 versus 12.9 MME). Both Clusters 2 and 10 had higher drug-related event rates after taper initiation than cluster 1 (mean 0.116 and 0.128 versus 0.074), higher mental health event rates (mean 0.089 and 0.075 versus 0.058), and higher death rates (mean 0.079 and 0.098 versus 0.036). The slow trajectory for cluster 1 and the low or zero doses in clusters 2 and 10 continued into the 15th month among patients with longer follow-up (See S1 Fig).

**Table 3:** Adverse events after taper initiation.

Cluster	No. patients	Drug-related	No. Excess drug-	Mental Health	No. Excess Mental	Deaths	No. Excess
		**events/1000**	**related events**	**events/1000**	**Health events**	**per 1000**	**Deaths**
		**(95%:LCI,UCI)**	**(95%:LCI,UCI)**	**(95%:LCI,UCI)**	**(95%:LCI,UCI)**	**(95%:LCI,UCI)**	**(95%:LCI,UCI) **
1	16,965	74(70,78)	-320(-387,-253)	58(55,62)	-240(-300,-180)	36(33,39)	-330(-377,-282)
2	13,025	116(111,122)	304(232,375)	89(85,94)	221(157,284)	79(74,84)	306(246,367)
10	531	128(100,156)	19(4,34)	75(53,98)	–	98(73,123)	23(9,36)
3 - 5*	185	97(55,140)	–	146(95,197)	14(4,23)	86 (46,127)	–
6 - 9*	226	80(44,115)	–	93(55,131)	–	–	–

^*^Clusters 3-5, clusters 6-9: results combined to adhere to small cell size policy, based on observed similarity of tapering patterns. ^–^Values masked due to small cell size policy (less than 11).

**Fig 3:**
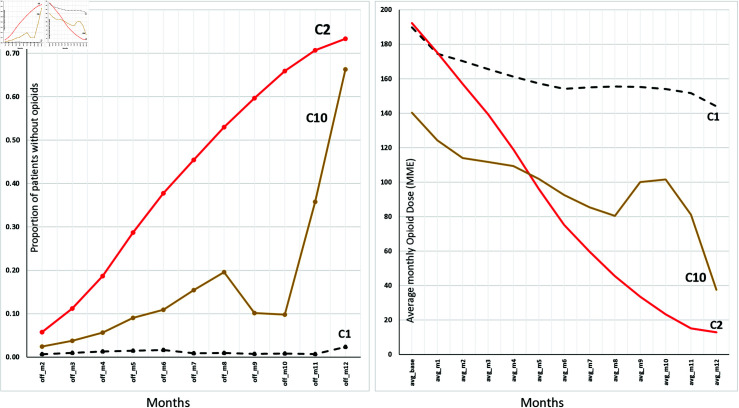
The proportion of patients without opioids, i.e., with an average monthly dose of 0 MME, in the three most populated clusters and their corresponding tapering trajectories.

The left panel in Fig 3 shows the proportion of patients in each of the 3 most populated clusters who received 0 MME dose in each month, while the right panel shows the corresponding mean dose trajectories. These results show that cluster 2 had the highest proportion of patients (73%) completely tapered off opioids at the end of 12 months, compared with clusters 10 (66%) and 1 (2%). Specifically, cluster 2 had a steep yet steady upward trend in the proportion of patients who were taken off opioids, whereas patients in cluster 1 almost uniformly stayed on opioids, and cluster 10 demonstrated a pattern of delayed discontinuation.

The remaining patients grouped into seven smaller clusters, all of which had patients who were tapered to or close to 0 MME. The pattern in Clusters 3, 4, and 5, a sudden drop from high to negligible doses, appears unlikely to be intentional and may be due to delayed prescription pick-ups or refills or errors in the submission or processing of pharmacy claims. Clusters 6, 7, 8, and 9 show a pattern of attempted gradual tapering over 6-11 months, but the taper was largely reversed and the subsequent trajectory was truncated due to the cohort design (See S2 Fig). In aggregate, these 4 clusters had drug-related event rates similar to cluster 1, but their mental health event rate was nearly twice that of cluster 1 (mean 0.093 versus 0.058) and similar to that of cluster 2. The number of deaths in clusters 6 through 9 was masked to adhere to small cell size policy (values less than 11).

## Discussion

In this large, diverse longitudinal cohort of patients with chronic pain receiving high dose opioids, unsupervised ML revealed substantial heterogeneity in dose tapering patterns, including the final opioid dose and post-tapering reversals. Spectral clustering revealed notable variation in the velocity and duration of tapering, post-tapering minimum doses, and subsequent re-initiation (taper reversal) of moderate-to-high opioid doses. The largest cluster (cluster 1) was characterised by very slow, gradual tapering from a mean baseline dose of 190 MME to 144 MME at 12 months, whereas the second largest cluster (cluster 2) was characterised by steep tapering from a mean baseline dose of 192 MME to only 12.9 MME (with 73% of patients discontinued). The latter cluster had a substantial excess of adverse events, consistent with previous findings [11,12,49,50] that rapidly tapering patients accustomed to high-dose prescription opioids may be associated with health risks. Our results suggest that there is a significant population of patients receiving high-dose opioids who may not tolerate tapering to very low doses; some of these patients in our cohort may have had undiagnosed opioid use disorders.

Clustering algorithms that are robust to outliers, such as spectral clustering, group rare events into small, sparse clusters [51]. The population that showed substantial dose reversal was about 1% of the total tapered population, which indicates that this phenomenon is rare but clinically important. These small clusters were characterised by rapid tapering to negligible doses, followed by re-initiation of opioids at moderately high doses. Taper reversals merit further exploration as they may be a marker of unsuccessful tapering followed by implementation of a more patient-centred care strategy. Previous studies have associated rapid tapering to very low doses with subsequent termination of ambulatory care [52], decreased adherence to medications for diabetes and hypertension [53], and disenrollment from health plans [54]. Our findings in clusters 6-9 suggest that clinicians may be able to safely resume prior opioid doses for some patients with worsening pain. It would be important to identify the factors that trigger taper reversal and to estimate subsequent adverse outcomes, considering the velocity of tapering along with the post-tapering final dose.

Since our aim was to discover novel patterns, the way to implement this is unsupervised machine learning with the fewest assumptions imposed on the data structure such as the number of clusters, cluster shape, complete and large separation versus overlapping clusters, sparse vs dense clusters, etc. Other authors have identified opioid dose trajectories using group-based trajectory modelling (GBTM) [54] or k-means clustering [55]. The analyses grouped patient-trajectories into simple groups such as decreasing, stable or increasing, etc., but they did not look for taper reversals, suggesting that all trajectories were maintained over time. On the other hand, Hayes and colleagues used GBTM on a national cohort of US veterans and identified nine tapering trajectories over 2 years, including one reversal trajectory [56]. However, GBTM is a supervised learning method that requires the user to select the number of trajectory-groups based on strong within-group similarity assumptions. Hence, model misspecifications will lead to wrong conclusions [35]. Given the size and heterogeneity of our cohort, we did not impose any restrictions on the number of subpopulations (clusters) or patient characteristics to be identified in the data. In addition to being fully data-driven, our analysis focused on patients for whom a taper had been initiated, to understand underlying heterogeneity in this clinically important subpopulation. Therefore, our results support and facilitate future analyses including comparing the outcomes of different tapering approaches with the alternative of not tapering at all.

Spectral clustering highlighted the taper reversal pattern along with other patterns already heavily reported in the literature. This finding confirms that spectral clustering can uncover important patterns that can be easily buried by larger signals. Spectral clustering’s main attractions are not assuming the shapes of clusters and its suitability for extremely large datasets [26,45]. However, we did not use cluster metrics such as silhouette scores to assess cluster quality because silhouette scores primarily favour spherical clusters [57,58]. For argument’s sake, let’s assume that our cluster boundaries are incorrect. Since our goal is not prediction but novel patterns detection, which was confirmed in the independent study by Hayes et al. [56], we are not concerned about cluster membership. This is why we used an unsupervised learning method as opposed to a supervised clustering method [15]. Finally, cluster metrics are also computationally intensive calculations and must be well-justified. Different metrics have different data assumptions and definitions of “good clusters”. The definition of good clusters is dependent on the objective of the scholar and the assumptions of the underlying data structure. Cluster metrics are valuable when the aim is to use the clusters as a tool for prediction, ranking, etc. However, in our case, the goal was to identify novel patterns that could be further investigated if found clinically meaningful. Lastly, in order to boost the overall cluster quality, given an optimisation criterion, even the best clustering algorithm might produce a few clusters with low quality and one way to reduce these poor-quality clusters is to avoid restricting or specifying the number of clusters [45].

We acknowledge limitations such as unknown intent of the prescribing provider. For example, the physician’s choice of a rapid or slow taper may be driven by unobserved characteristics of patients or their medical histories, which may independently contribute to the resulting outcomes. Finally, the current data do not capture illicit opioid use, sharing of opioids, or methadone administered in certified treatment programmes. We also acknowledge the need for an updated cohort, given recent decreases in opioid prescribing [8], although the rate of overdose deaths involving prescription opioids has only decreased about 12% from a peak in 2021, after a significantly increasing trend from 2002 to 2016 [59]. The taper reversal can be investigated using rare event models in richer prospective cohort data that include information from electronic health records.

Our analysis is still relevant to current clinical treatments and opioid trends because opioid dispensing rates remain very high in states that are highly represented in our data. It is clear from epidemiological data that the United States has made relatively little progress in reducing overdoses from prescription opioid analgesics, so the phenomenon of taper reversal remains highly relevant for both patient and population health. Since our analysis was performed using a large dataset that is representative of the commercially and Medicare-insured population of the United States, these results are generalisable to those populations. AI is a suitable tool for understanding rare events because these events do not follow normal distributions nor can their distribution be pre-specified. Dose tapering to discontinuation may plausibly increase the risk of subsequent opioid overdose if these opioid-dependent patients seek alternative opioids from illicit sources or mix opioids with other sedating drugs such as benzodiazepines, thereby negating the purpose of dose tapering. Reversal of tapering may be an important signal; future research should explore whether these events are preceded by pain-related ED or urgent care visits, changes of provider, increasing patient-reported pain scores, or discontinuation of nonopioid analgesics. Our results, obtained using an unsupervised AI approach, may be sufficiently compelling to warrant further investigations into dose tapering patterns, including multiple and reversed tapers, to inform future prescribing policies and clinical practice, and to guide the design of clinically useful predictive models.

## Supporting information

S1 Table Taper Trajectories. Table showing the average monthly dose of the 10 cluster-trajectories.
(XLSX)

S1 Fig Graph of Tapering up to 15 months. Graph showing the Taper Trajectories up to 15 months.
(DOCX)

S2 Fig Graph of Taper Reversal Trajectories. Taper Reversal Trajectories up to 12 months for the four small clusters.
(DOCX)
