# Supplementary figures and images for: Unsupervised machine learning identifies opioid taper reversal patterns in a longitudinal cohort (2008–2018)
Source: PLOS Digit Health. 2025 Apr 7;4(4):e0000785. doi: 10.1371/journal.pdig.0000785 (PMC11975097; doi:10.1371/journal.pdig.0000785)

**
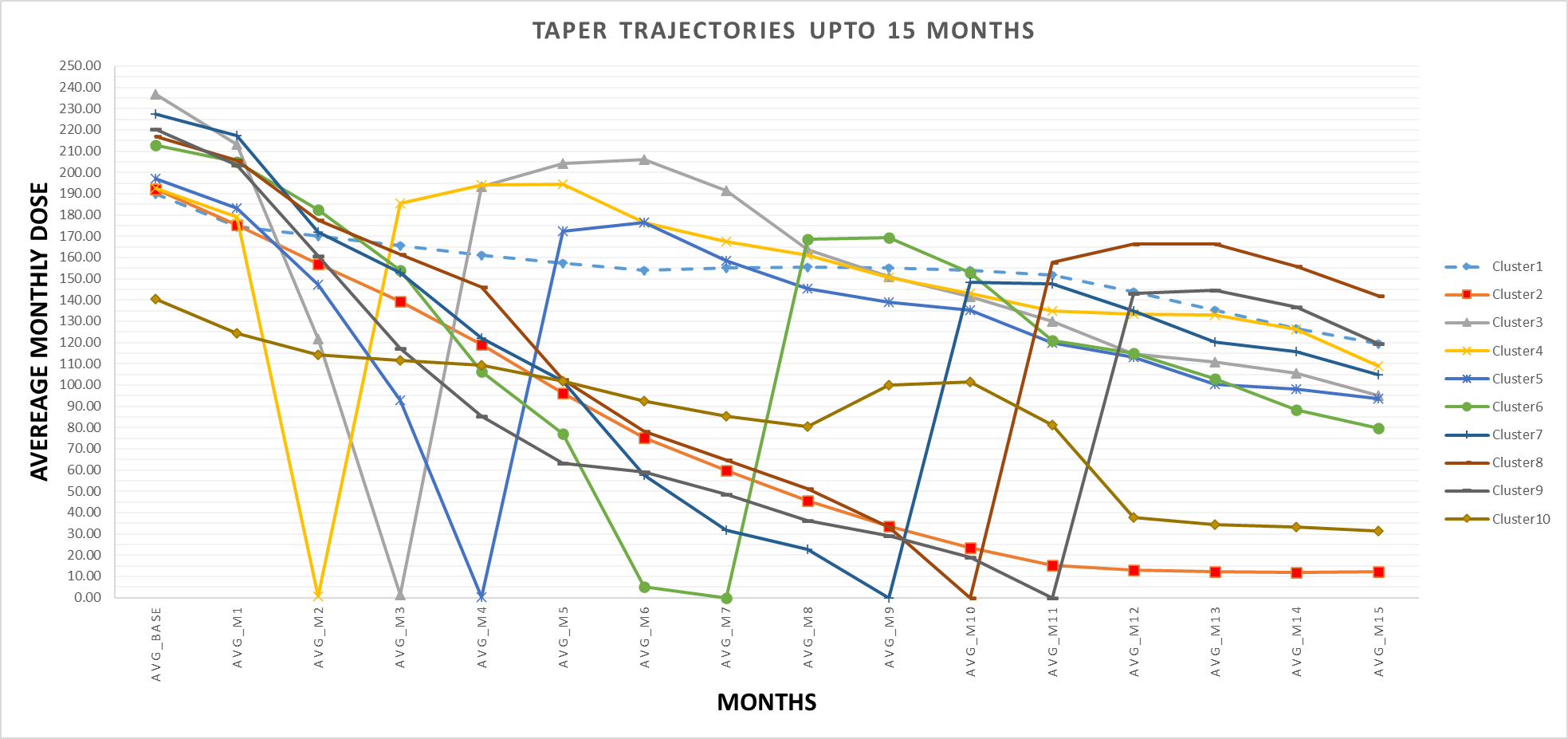
**

**S1 Fig**: This shows the taper trajectories until 15 months for all clusters

Supplement: S1 Fig — Graph showing the Taper Trajectories up to 15 months. (DOCX) [file pdig.0000785.s002.docx]
