# Supplementary material for: Unsupervised machine learning identifies opioid taper reversal patterns in a longitudinal cohort (2008–2018)
Source: PLOS Digit Health. 2025 Apr 7;4(4):e0000785. doi: 10.1371/journal.pdig.0000785 (PMC11975097; doi:10.1371/journal.pdig.0000785)
Supplement: S2 Fig — Taper Reversal Trajectories up to 12 months for the four small clusters. (DOCX) [file pdig.0000785.s003.docx]

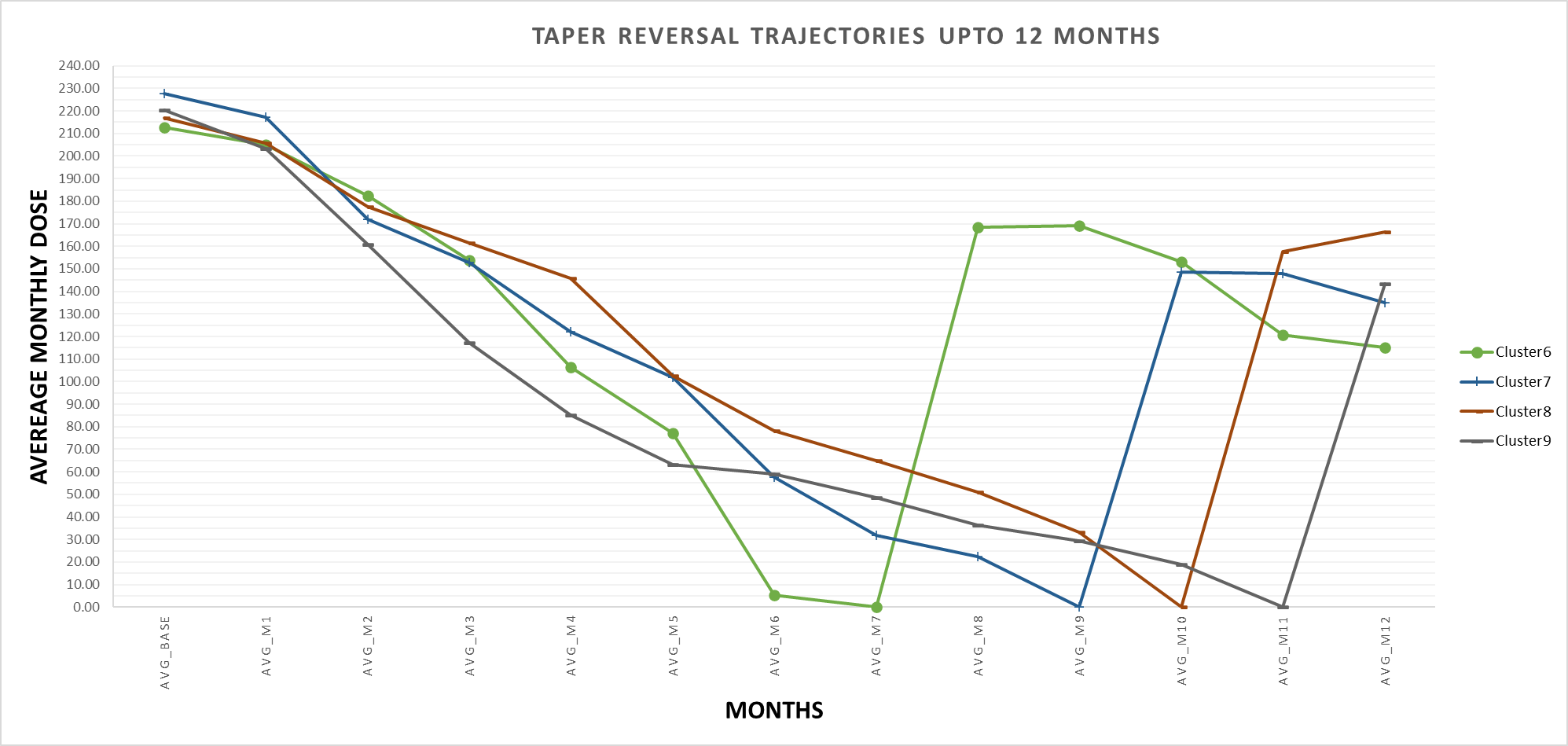


**S2 Fig**: This shows the taper trajectories of the 4 small clusters where patients underwent a taper reversal
